# Pathogenic *Escherichia coli*, *Salmonella* spp. and *Campylobacter* spp. in Two Natural Conservation Centers of Wildlife in Portugal: Genotypic and Phenotypic Characterization

**DOI:** 10.3390/microorganisms10112132

**Published:** 2022-10-27

**Authors:** Angela Pista, Leonor Silveira, Sofia Ribeiro, Mariana Fontes, Rita Castro, Anabela Coelho, Rosália Furtado, Teresa Lopes, Carla Maia, Verónica Mixão, Vítor Borges, Ana Sá, Vanessa Soeiro, Cristina Belo Correia, João Paulo Gomes, Margarida Saraiva, Mónica Oleastro, Rita Batista

**Affiliations:** 1 National Reference Laboratory for Gastrointestinal Infections, Department of Infectious Diseases, National Institute of Health Doutor Ricardo Jorge, Avenida Padre Cruz, 1649-016 Lisbon, Portugal; 2 Food Microbiology Laboratory, Food and Nutrition Department, National Institute of Health Doutor Ricardo Jorge, Avenida Padre Cruz, 1649-016 Lisbon, Portugal; 3 Food Microbiology Laboratory, Food and Nutrition Department, National Institute of Health Doutor Ricardo Jorge, Rua Alexandre Herculano 321, 4000-055 Porto, Portugal; 4 Genomics and Bioinformatics Unit, Department of Infectious Diseases, National Institute of Health Doutor Ricardo Jorge, Avenida Padre Cruz, 1649-016 Lisbon, Portugal; 5 Tapada Nacional de Mafra, Portão do Codeçal, 2640-602 Mafra, Portugal; 6 Centro de Recuperação do Parque Biológico de Gaia, Rua da Cunha, Avintes, 4430-812 Vila Nova de Gaia, Portugal; 7 Faculty of Veterinary Medicine, Lusófona University, Campo Grande 376, 1749-024 Lisbon, Portugal

**Keywords:** Pathogenic *Escherichia coli*, *Salmonella* spp., *Campylobacter* spp., Whole-genome sequencing, wild animals, Portugal

## Abstract

Human–wildlife coexistence may increase the potential risk of direct transmission of emergent or re-emergent zoonotic pathogens to humans. Intending to assess the occurrence of three important foodborne pathogens in wild animals of two wildlife conservation centers in Portugal, we investigated 132 fecal samples for the presence of *Escherichia coli* (Shiga toxin-producing *E. coli* (STEC) and non-STEC), *Salmonella* spp. and *Campylobacter* spp. A genotypic search for genes having virulence and antimicrobial resistance (AMR) was performed by means of PCR and Whole-Genome Sequencing (WGS) and phenotypic (serotyping and AMR profiles) characterization. Overall, 62 samples tested positive for at least one of these species: 27.3% for STEC, 11.4% for non-STEC, 3.0% for *Salmonella* spp. and 6.8% for *Campylobacter* spp. AMR was detected in four *E. coli* isolates and the only *Campylobacter coli* isolated in this study. WGS analysis revealed that 57.7% (30/52) of pathogenic *E. coli* integrated genetic clusters of highly closely related isolates (often involving different animal species), supporting the circulation and transmission of different pathogenic *E. coli* strains in the studied areas. These results support the idea that the health of humans, animals and ecosystems are interconnected, reinforcing the importance of a One Health approach to better monitor and control public health threats.

## 1. Introduction

Wildlife–livestock–human interfaces represent critical points for cross-species interaction and consequent transmission and emergence/re-emergence of pathogens into new host populations [1]. In fact, more than 1400 species of human pathogens have been recognized, of which more than half are of zoonotic origin. These pathogens can have a broad range of hosts, and it is a complex task to assess direct and indirect economic losses in sectors such as public health, animal health, and the environment [2,3,4]. As such, the implementation of integrative wildlife surveillance programs, following a One Health approach, is critical to learn about the interconnectedness of different zoonotic pathogens present in humans, animals and the environment and, consequently, to enhance the detection and control of public health threats [5,6].

According to the Zoonoses Directive 2003/99/EC, it is mandatory for European Union (EU) Member states (MS) to collect and report relevant data on zoonoses, zoonotic agents, antimicrobial resistance (AMR) and foodborne outbreaks to the European Food Safety Authority (EFSA). In 2020, zoonoses data from 36 European countries (27 MS and 9 non-MS) were reported. The analysis of the results showed that campylobacteriosis and salmonellosis were the most common reported zoonoses, with the total number of confirmed human cases being stable between 2015 and 2020. Shiga toxin-producing *Escherichia coli* (STEC) was the fourth most reported foodborne gastrointestinal infection in humans. Regarding STEC, after an increase of cases from 2015 to 2019, a reduction of infections was observed in 2020, probably due to the impact of the restrictions imposed by the COVID-19 pandemic [4].

Nowadays, there is evidence that wild animals are important reservoirs in the transmission of zoonotic agents, both to other animals and to humans. Wild boars (*Sus scrofa*) have a worldwide geographic distribution, with a significant increase in Europe in the last decades [7]. In Portugal, wild boars and deer (red deer, *Cervus elaphus*; fallow deer, *Cervus dama*) are mainly found in limited and controlled areas. In general, both species of these wild animals are considered to be potential vectors in the transmission of severe pathogens to humans, either by direct contact, for instance, in the context of hunting activities, or by indirect contamination of foodstuffs, water or environmental areas frequented by people or pets, via urine or feces. Commensal and pathogenic strains may also represent a serious hazard and an important risk for human health, regarding their antimicrobial resistance [8].

Although some studies which have focused on wild animals as reservoirs for presumptive zoonoses have been published in Europe, from a One Health perspective, they are scarce and have mainly focused on one pathogen [9,10,11,12,13,14,15,16,17,18,19,20].

The main goals of this study were to assess the occurrence of pathogenic *E. coli*, *Salmonella* spp., and *Campylobacter* spp. in wild animals (wild boar, red deer, fallow deer, hedgehogs (*Erinaceus europaeus)*, and genets (*Genetta genetta*)) from two different populations and habitats, and to characterize the serotypes, the associated virulence markers, the antimicrobial resistance (AMR) profiles and the genetic diversity of the obtained isolates. Furthermore, as wild animals can act as efficient AMR reservoirs, as well as sources of epidemiological information and potential links between strains from human, livestock and natural environments, determination of the AMR profile of a fraction of the non-pathogenic *E. coli* was carried out. In addition, a comprehensive genomic study targeting pathogenic *E. coli* strains was performed.

To our best knowledge, this is the first study in Portugal, and one of the few in Europe, that simultaneously evaluates the presence of, and characterizes, *E. coli* (STEC and non-STEC), *Salmonella* spp. and *Campylobacter* spp. in wildlife, using both culture dependent methodologies and Whole-Genome Sequencing (WGS).

## 2. Materials and Methods

### 2.1. Study Areas

The selected sampling areas were two natural wildlife conservation centers, located in distinct geographical regions: Tapada Nacional de Mafra (TNM), in the Lisbon district, and The Centro de Recuperação de Fauna do Parque Biológico de Gaia (CRFPBG), in the Oporto district. TNM is a protected green area of about 1200 hectares, home to free-roaming wild boar and deer, among other animals. It is a national hunting zone (in a controlled and limited way) and used for rural tourism and leisure. A 17.5-km-long river, which originates in a nearby village, crosses through TNM to flow out into the Atlantic Ocean. CRFPBG is a 35-ha park located in an agroforestry area, and shelters hundreds of species in their natural habitats. The CRFPBG recuperates wild animals that have suffered any form of injuries or which were illegally held in captivity.

### 2.2. Study Population and Sample Collection 

Animal fecal samples (*N* = 132) analyzed in this study were collected between July 2020 and June 2021, in a non-controlled sampling. The sampling was conducted with disposable sterile tools and the samples placed in sterile stool sample containers. Fresh feces of wild boar, red deer and fallow deer were collected at TNM (*N* = 113), under the supervision of a local biologist. Collection points were spaced apart to increase the likelihood of screening different individuals. Due to the COVID-19 pandemic restrictions, sampling was carried out in July, October, and November 2020, and later in May 2021. Feces of hedgehogs and genets were collected from animals rescued by CRFPBG (*N* = 19) between November 2020 and June 2021, excluding December 2020, and February and March 2021. All samples were kept refrigerated and processed immediately after arriving at the laboratory.

### 2.3. Isolation Methodology

For *E. coli*, a pre-enrichment in Buffered Peptone Water (1:10 dilution; BPW-Oxoid, Basingstoke, Hampshire, UK) was performed at 37 °C overnight and seeded in Tryptone Bile X-Glucuronide (TBX, Biokar Diagnostics, Pantin, France) and CHROMagar STEC (CHROMagar, Paris, France) agar plates for 20 h at 44 °C and 24 h at 37 °C, respectively. Five to ten colonies were selected and sub-cultured on Tryptone Soy Agar (TSA, Biokar Diagnostics) overnight at 37 °C. Suspicious colonies were confirmed by biochemical identification on the VITEK 2 compact system (bioMérieux, Marcy L’Etoile, France) or by amplification of *E. coli* 16S rRNA, according to Sabat et al. (2000) [21]. All positive isolates were stored at −80 °C in Tryptone Soy Broth (TSB, Biokar Diagnostics) with 20% glycerol. 

Isolation of *Salmonella* spp. was achieved according to ISO 6579-1:2017 [22]. After a pre-enrichment step, as previously described, a selective enrichment of 1 mL culture was performed in 10 mL of Muller–Kauffmann tetrathionate–novobiocin broth (MKTTn, Biokar Diagnostics) at 37 °C for 24 h. Simultaneously, 50 µL were added to the center of a Modified Semi-solid Rappaport–Vassiliadis agar plate (MSRV, Biokar Diagnostics) and incubated at 41.5 °C for 24 h. Finally, IRIS *Salmonella* agar (Biokar Diagnostics) was used for plating-out for 24 h at 37 °C, and colonies of presumptive *Salmonella* spp. were isolated on TSA at 37 °C for 24 h. The identity of suspicious isolates was confirmed by biochemical identification on the VITEK 2 compact system. All positive isolates were stored at −80 °C, as previously described.

*Campylobacter* spp. isolation was based on ISO 10272-1:2006-1 [23]. A test portion of about 1 g was added to 10 mL of Bolton broth enrichment medium (Bolton Broth with Bolton broth selective supplement, and 5% Horse Blood Lysed, Oxoid) and incubated in a microaerobic atmosphere at 37 °C for 4–6 h and, then, at 41.5 °C for 48 h. Plating-out was performed by applying five drops (≈100 µL) of the enrichment culture to a 0.65-µm pore-size filter (Nitrocellulose membrane filters, Whatman, Little Chalfont, Buckinghamshire, UK) placed over a plate of Columbia Agar + 5% Sheep blood (COS; bioMérieux). The filter was left in contact with the surface of the agar for 15 min at room temperature. Furthermore, about 100 µL of the enrichment culture was spread over a plate of modified Charcoal Cefoperazone Deoxycholate agar (mCCDA, Oxoid). Plates were incubated in a microaerobic atmosphere at 41.5 °C for 48 h. Three to five presumed *Campylobacter* colonies were sub-cultured on COS and their identification confirmed by oxidase activity and MALDI-TOF mass spectrometry (VITEK^®^ MS, bioMérieux). All positive isolates were stored, as previously described. 

### 2.4. Bacterial Typing and Antimicrobial Susceptibility Testing

Identification of potentially pathogenic *E. coli* was performed by screening for the presence of *eae*, *aggR*, *elt*, *estp*, and *ipaH* virulence genes (VG) by multiplex PCR (modified from Persson 2007, Boisen 2012, and Fujioka 2013) [24,25,26] and for the presence of Shiga toxins *stx1* and *stx2* [27]. Detection was initially performed in pools with a maximum of 10 colonies from each sample. Whenever a PCR-positive result was detected, all the pool isolates were analyzed individually. A single colony was boiled for 10 min, cooled for 1 min, centrifuged at 16.200× *g* for 10 min, and the supernatant was used as a DNA template. The amplification of VG was performed in a reaction volume of 25 µL and using the HotStar Taq master mix (Qiagen, Hilden, Germany), according to the manufacturer’s recommendations. For Shiga toxins detection, two separate reactions with a final volume of 20 µL and using the HotStar Taq master mix were prepared. Primers and PCR profile are presented in the Supplementary Material Table S1. Strains of External Quality Assessments (organized by the Statens Serum Institut, Copenhagen, Denmark) were used as positive controls. Amplicons were visualized on a 2.5% agarose gel in 0.5x TBE buffer at 100 V for 30–45 min. An *E. coli* isolate was classified as potentially pathogenic (STEC; EPEC, Enteropathogenic *E. coli*; EAEC, Enteroaggregative *E. coli*; ETEC, Enterotoxigenic *E. coli*; EIEC, Enteroinvasive *E. coli*) when at least one of the tested genes was detected.

Antimicrobial susceptibility testing (AST) was performed according to the Kirby–Bauer method in all pathogenic *E. coli* (*N* = 52) and in 42.3% (*N* = 33) of the non-pathogenic *E. coli* isolates (total of 85 isolates), following the European Committee on Antimicrobial Susceptibility Testing recommendations [28]. For the testing, the following panel of 18 antimicrobials was used: Ampicillin (AMP), Amoxicillin-Clavulanic Acid (AMC), Azithromycin (AZM), Cefepime (FEP), Cefotaxime (COX), Cefoxitin (FOX), Ceftazidime (CZD), Ceftriaxone (CRO), Chloramphenicol (CHL), Erythromycin (ERY), Gentamicin (GMN), Meropenem (MEM), Nalidixic Acid (NAL), Ciprofloxacin (CIP), Sulfamethoxazole (SMX), Tetracycline (TET), Tigecycline (TGC), and Trimethoprim (TMP). The results were interpreted according to the EUCAST epidemiological cut-off values (ECOFFs) [28]. An isolate was classified as multidrug-resistant (MDR) when it presented resistance to three or more antimicrobial classes.

*Salmonella* isolates were serotyped by the slide agglutination method for O and H antigens (SSI Diagnostica, Hillerod, Denmark; Sifin diagnostics, Berlin, Germany), according to the Kauffmann–White–Le Minor scheme [29]. AST was performed, as previously described, for *E. coli* to 17 antimicrobials, including Pefloxacin (PEF) instead of CIP, and excluding ERY.

For *Campylobacter* spp., AST was conducted as previously described for CIP, ERY, TET, GMN according to EUCAST 2021 [29], and for AMP and AMC according to Comité de l’antibiogramme de la Société Française de Microbiologie [30]. 

### 2.5. Whole-Genome Sequencing, in Silico Typing and Screening of Virulence/AMR Genes

Genomic DNA was extracted from fresh cultures of all the pathogenic isolates using the ISOLATE II Genomic DNA Kit (Bioline, London, England, UK), and quantified in the Qubit fluorometer (Invitrogen, Waltham, MA, USA) with the dsDNA HS Assay Kit (Thermo Fisher Scientific, Waltham, MA, USA), according to the manufacturer’s instructions. *Salmonella enterica enterica* ser. Veneziana and *Campylobacter hyointestinalis* isolates were not sequenced, as they are currently not considered to be relevant pathogens in Portugal. DNA was then subjected to the NexteraXT library preparation protocol (Illumina, San Diego, CA, USA) prior to cluster generation and paired-end sequencing (2 × 250 bp or 2 × 150 bp) on either a MiSeq or a NextSeq 550 instrument (Illumina), according to the manufacturer’s instructions. FastQC v0.11.5 (https://www.bioinformatics.babraham.ac.uk/projects/fastqc/ (accessed on 6 October 2022)) was used for quality control and Trimmomatic v0.38 [31] for trimming low-quality bases. For *E. coli* and *Salmonella*, sequencing reads were submitted to the Center for Genomic Epidemiology web server (https://cge.cbs.dtu.dk (accessed on 6 October 2022)) for identification of antimicrobial resistance genes (ResFinder 4.1), *in silico* Multilocus Sequence Typing (MLST) (MLST 2.0), *E. coli* virulence genes identification (VirulenceFinder 2.0), and *in silico E. coli* (SerotypeFinder 2.0) and *Salmonella* serotyping (SeqSero 1.2). For *Campylobacter*, *in silico* MLST was determined at the PubMLST platform (https://pubmlst.org/ (accessed on 6 October 2022)).

Sequencing reads were deposited in the European Nucleotide Archive (ENA) under the bioproject PRJEB54735 (*E. coli*), PRJEB32515 (*Salmonella*) and PRJEB46733 (*C. coli*). Accession numbers for each isolate are listed in Supplementary Material Table S2. 

### 2.6. Core-Genome MLST Clustering Analysis of Pathogenic E. coli 

Core-genome MLST (cgMLST) analysis was performed for the 52 pathogenic *E. coli* isolates. To this end, we performed *de novo* genome assembly with the INNUca pipeline v4.2.2 (https://github.com/B-UMMI/INNUca (accessed on 6 October 2022)) [32] using default parameters. *De novo* genome assembly was performed with SPAdes v3.14 [33]. Reads were aligned with Bowtie v2.2.9 [34] and the assembly was polished with Pilon v1.23 [35]. Species confirmation/contamination screening was performed with Kraken2 v2.0.7 [36]. As the sample Ec-TM86 revealed traces of contamination with *Morganella morganii*, the *E. coli*-classified contigs were retrieved with an *in-house* script (https://github.com/vmixao/scripts/kraken_results2filter_assembly.py (accessed on 6 October 2022)), yielding a final assembly with the expected genome size for *E. coli*.

Allele-calling was performed with chewBBACA v2.8.5 [37] using the latest version of the 7601-loci wgMLST schema, curated and available at Chewie-NS website (https://chewbbaca.online/, last change date on 31 May 2021) [38]. First, a global clustering analysis was performed with ReporTree v1.0.0 (https://github.com/insapathogenomics/ReporTree (accessed on 6 October 2022)) [39] over the core-genome of the dataset (i.e., a sub-schema comprising loci called in all samples, *n* = 2567 loci), using GrapeTree (MSTreeV2 method) [40]. Clusters of closely related isolates were determined at a distance threshold equivalent to 0.34% of the total number of alleles of the applied cgMLST schema (i.e., 0.0034 × 2567 loci = ~9 allelic differences - ADs), as this threshold can provide a proxy to the identification of genetic clusters with potential epidemiological concordance (i.e., “outbreaks”) for *E. coli* [32]. Of note, the choice of a loci panel called in 100% of the isolates for this first clustering analysis, and resulted from preliminary analyses showing that a more flexible threshold for core-schema definition (including loci that were only called in 95% and 98% of samples) did not result in a substantial increase of the core-genome size, or, thus, in resolution. The schema size was also not impacted by the inclusion/exclusion of the curated Ec-TM86 assembly. Second, an in-depth clustering analysis for each ST was performed using a ST-specific core-genome schema (i.e., a sub-schema comprising the loci called in all the isolates of the same ST) and applying the same threshold for cluster definition. By maximizing the shared genome, this dynamic approach allowed increasing the resolution power and confidence concerning the initially detected clusters. Summary reports for the determined genetic clusters was automatically generated by ReporTree v1.0.0 [39], including cluster composition, timespan and distribution of host species or AMR phenotypes. In order to assess the genetic differences at SNP level between the isolates Ec-TM26 and Ec-TM69, which presented different AMR phenotypes but similar allelic profiles, an additional SNP analysis was conducted using Snippy v4.6.0 (https://github.com/tseemann/snippy (accessed on 6 October 2022)), setting “--mapqual 20 --mincov 10 --minfrac 0.51 --basequal 20” and using the Ec-TM26 assembly as reference. 

## 3. Results

### 3.1. Detection and Characterization of Isolates

In the present study, 132 fecal samples from wild animals (51 from wild boar, 50 from fallow deer, 12 from red deer, 18 from hedgehogs, and 1 from a genet) were evaluated for the presence of pathogenic *E. coli* and *Salmonella* spp., and 118 samples for *Campylobacter* spp. (39 wild boar, 50 fallow deer, 12 red deer, 16 hedgehog, and 1 genet) (Table 1). 

Overall, 62 of the 132 tested samples (47.0%) tested positive for at least one of the evaluated pathogenic bacterial species. In total, pathogenic *E. coli* (STEC and non-STEC), *Salmonella* spp. and *Campylobacter* spp. were detected in 37.9% (50/132), 3.0% (4/132) and 6.8% (8/118) of the samples, respectively (Table 1). There were no samples presenting two different pathogenic bacterial species simultaneously.

Pathogenic *E. coli* was recovered in 17.6% (9/51) of the wild boar samples, 68.0% (34/50) of fallow deer samples, 50% (6/12) of red deer samples, and 5.5% (1/18) of the hedgehog samples (Table 1). STEC was the most frequently detected pathotype (27.3%; 36/132), being identified in wild boar, fallow deer and red deer samples, followed by EPEC (9.1%; 12/132), identified in wild boar, fallow deer, red deer and hedgehog samples, and ETEC (2.3%; 3/132), found only in wild boar samples (Table 1). Two fallow deer samples contained two different pathogenic *E. coli* isolates: one sample contained one EPEC and one STEC isolate, and the other contained two different STEC isolates (Ec-TM29 and EcTM30, respectively; Table S2). 

Although *Salmonella* was rarely found in the studied animals, it is noteworthy that there was the identification of three distinct serovars: two cases of *S.* Schleissheim (1.5%; 2/132), recovered from wild boar and red deer samples, one *S.* Enteritidis (0.8%; 1/132) recovered from a wild boar sample, and one *S*. Veneziana (0.8%; 1/132) isolated from a hedgehog sample (Table 1). 

Regarding *Campylobacter*, *C. hyointestinalis* was detected in seven wild boar samples (5.9%; 7/118) and *Campylobacter coli* in one genet’s sample (0.8%; 1/118) (Table 1). 

### 3.2. Antimicrobial Susceptibility Testing and in Silico Genotyping

The *S*. Schleissheim isolates belonged to ST53 and *S*. Enteritidis to ST11. All *Salmonella* isolates were phenotypically susceptible to the 17 tested antimicrobials. WGS of the *S*. Schleissheim and *S*. Enteritidis, revealed the presence of the *aac(6′)-Iaa* gene.

The *Campylobacter coli* isolate was identified as belonging to ST1595. The isolate was resistant to CIP, TET and AMP, and harbored the corresponding resistance determinants, the *gyrA* (Thr86Ile) mutation, and the *tetO* and *blaOXA-61* genes, respectively. 

Among the 52 *E. coli* pathogenic isolates, 15 O antigens and 10 H antigens were identified *in silico*. The most prevalent serotypes were O146:H21 and O75:H8 (10 STEC isolates each), followed by serotypes O146:H28 (8 STEC isolates) and O27:H30 (5 STEC isolates) (Table 2).

*E. coli* MLST analysis identified 16 ST, with ST13 being the most common (11 isolates) followed by ST442 (10 isolates), ST738 (8 isolates) and ST753 (5 isolates) (Table 2).

For *E. coli*, the antimicrobial resistance level was low, being phenotypically detected in 4.7% of the tested isolates (4/85; 5.8% in pathogenic and 3.0% in non-pathogenic *E. coli* isolates), of which one was classified as MDR. *In silico* genotyping confirmed the found pathogenic *E. coli* AMR phenotypes (Table S2). One wild boar ETEC isolate (Ec-TM17) was resistant to TET and SMX (*tet(B)* and *sul2* genes); one red deer EPEC isolate (Ec-TM100) was resistant to AMP, CIP and NAL (*blaTEM-1D*, *qnrB36*, *qnrB19*, *qnrB82*, *gyrA*), one MDR wild boar EPEC isolate (Ec-TM26) was resistant to AMP, CHL and SMX (*blaTEM-1B*, *floR*, *sul2*), and one non-pathogenic *E. coli* isolate (Ec-PBG26), recovered from the genet faecal sample, was resistant to SMX. 

All 52 pathogenic *E. coli* isolates contained virulence genes encoding toxins (Table 2), as well as the genes used for the primarily pathotype classification, e.g., by PCR. The most frequently detected genes were *ehxa* (76.9%), *stx2b* (71.2%), *subA* (59.6%), *mchF* (51.9%), and *ast A* (50.0%). Other genes are mentioned in Table 2 and Table S2. Among STEC isolates, 70.3% (26/37) presented only the *stx2* gene (*stx2b*) and 29.7% (11/37) the *stx1* and *stx2* genes (*stx1c* and *stx2b*), and all of them were *eae*-negative (Table 2, Table S2).

The three isolates encoding the major number of virulence factors were all EPEC isolates: Ec-TM26 (ST137; O145:HND; MDT), the Ec-TM57 (ST29; O70:H11), and the Ec-TM65 (ST29; O26:H11) (Table 2). 

### 3.3. Core Genome MLST Clustering Analysis of Pathogenic E. coli Isolates

**Figure 1:**
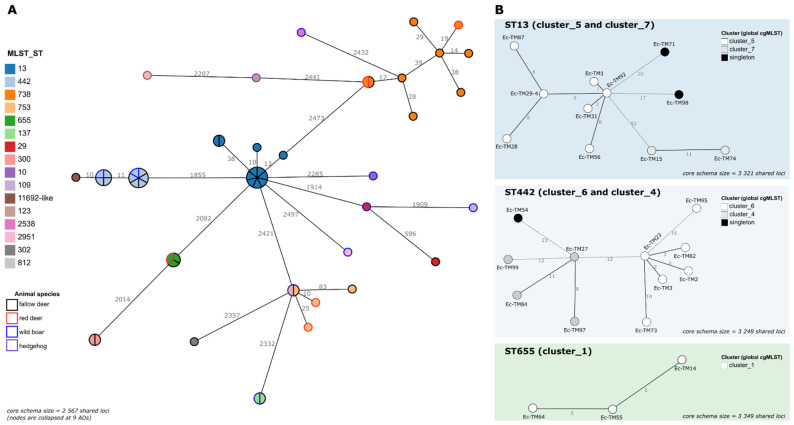
The cgMLST analysis of pathogenic *E. coli*. (**A**) The global Minimum Spanning Tree (MST) was reconstructed, based on the core-genome of the 52 isolates (i.e., a sub-schema of the original 7601-loci wgMLST schema comprising loci called in all samples). Each circle (node) represents a single isolate or a cluster of isolates linked by ≤9 ADs (corresponding to the 0.34% threshold applied for cluster investigation). Each division in a node corresponds to a single isolate. Node colors reflect the ST (inner color) and the animal species (outer line color) of each isolate. Lines connect nodes with ADs above the applied threshold, with the numbers representing the ADs between nodes. (**B**) For each ST of interest (details in Table 3), an MST was constructed, based on a dynamic cgMLST analysis, enrolling a maximized ST-specific core-genome schema (i.e., a sub-schema comprising the loci called in all the isolates of the same ST). Only the MSTs corresponding to STs harboring clusters with >2 isolates are presented (see details for the others in Table 3). Each circle (node) represents a unique allelic profile, with colors reflecting the genetic cluster to which the isolate belonged in the global cgMLST analysis. Straight and dotted lines reflect nodes linked with the ADs below/equal, and above, the 0.34% threshold applied for cluster investigation, respectively. The numbers in the connecting lines represent the ADs between isolates. All MSTs were constructed using the GrapeTree MSTreeV2 algorithm implemented in the ReporTree pipeline (https://github.com/insapathogenomics/ReporTree (accessed on 6 October 2022)), using the chewieNS schema (https://chewbbaca.online/ (accessed on 6 October 2022)). Data visualization was adapted from the GrapeTree dashboard.

A key step in One Health surveillance is the assessment of the genetic diversity of the circulating pathogens, including the identification of strains circulating among different host species and/or across long periods of time. To assess whether different *E. coli* pathogenic isolates (collected at different time points and/or sources) cluster together at high resolution level (thus, potentially representing the same circulating strain), a first cgMLST analysis comprising the 52 isolates was performed (Figure 1). 

A threshold of 0.34% ADs (9 ADs in 2567 shared loci) was applied to the generated MST as a proxy to identify clusters of very closely related isolates [32]. Despite the low number of samples present in our dataset, 57.7% (30/52) of the isolates integrated clusters, in a total of 9 genetic clusters (2 to 7 isolates per cluster) across seven STs and two pathotypes (Table 3). Six out of the nine genetic clusters comprised isolates collected from different animal species, but the largest cluster (cluster_5; STEC) was exclusively isolated from fallow deer (Figure 1, Table 3, Supplementary Material Table S2). Of note, the time span of most of the clusters extended almost up to four months.

To increase the clustering resolution, an additional cgMLST analysis per ST, maximizing the number of shared loci under evaluation, was performed. This in-depth assessment not only confirmed the detected clusters but also provided further clues about extra isolate/cluster linkages (Figure 1 and Table 3). Of particular note was a well-confirmed genetic cluster (cluster_9) comprising two EPEC isolates, collected from different animal species, with different antibiotic-resistance phenotypes (Table 3), one of them being susceptible (Ec-TM69) to the tested antimicrobials and the other (Ec-TM26) being resistant to AMP, CHL and SMX (Table 3, Supplementary Material Table S2). Finely comparative cgMLST and SNP analyses of these isolates confirmed their high genetic relatedness (≤5 ADs/SNPs), while suggesting horizontal gene transfer as the source of the Ec-TM26 resistance phenotypes. Indeed, AMP- and SMX-resistance could be linked to the presence of *blaTEM-1B* and *sul2* genes, respectively, both co-localizing in the same putative mobile genetic element, which also harbored the *aph(6)-Id* and *aph(3′’)-Ib* genes, linked to streptomycin resistance (phenotype not tested). The CHL resistance, associated with the presence of the *floR* gene, again fell into a putative mobile genetic element. Despite the near isogenicity of Ec-TM26 and Ec-TM69 in the extended core genome, these elements were confirmed to be absent in the susceptible Ec-TM69. 

## 4. Discussion

The occurrence of pathogenic *E. coli* in fecal samples from wild ungulates in Europe is extremely variable. The frequency value found in this study was in accordance with the ones reported in other studies done recently in Italy [10] and Poland [16], but higher than studies found in Spain [11] and Portugal [15]. Indeed, sometimes, even studies within the same country report different outcomes [18,41] and, in some cases, researchers have not found pathogenic *E. coli* in analyzed samples [42,43,44]. This heterogeneity may be related to numerous factors, such as the proximity of urban areas, and the number of animals per ha, or may even be related to the season in which sample collection was conducted. The high frequency of pathogenic *E. coli* found in TNM ungulates may be justified by the fact that the studied population is from a geographically limited area that is in close contact with humans (through visits and hunting activities) and with a high concentration of animals per ha.

For *Salmonella* and *Campylobacter*, the comparison of the results found in this study with other European studies performed in ungulate fecal samples shows highly variable outcomes. Regarding *Salmonella*, several studies have reported the absence of this pathogen in tested samples [18,42,43]. However, there are several other research works reporting frequency values between 1.1 and 10.8% [12,17,45,46,47,48], and at least one reporting a higher value (17.5%) in wild boars, particularly in populations co-habiting with cattle, where the rate increases to 35.7% [49]. In the case of *Campylobacter,* there are several studies reporting frequency values below 5% [17,42,43,50]. However, there is at least one study, in Spain, which referred to a rate of 15.2% in wild artiodactyl species [51] and another one, carried out in Italy, which reported a 91.66% frequency value of *C. coli* in wild boars [19].

As AMR poses a major worldwide threat to human health [52], the determination of the AMR profile of all pathogenic isolates, as well as of a part of the commensal *E. coli*, was carried out. In compliance with this study, the presence of several antimicrobial resistance genes had already been detected in other *E. coli*, *Salmonella* and *Campylobacter* isolates from fecal samples of wild ungulates in Europe [10,17,19,43,45,47,49,51]. In 2018 and 2019, in accordance with Commission Implementing Decision 2013/652/EU, phenotypic AMR was monitored in *E. coli* isolates obtained from fecal samples of the most relevant food-producing animals at slaughter (fattening pigs, calves under 1 year of age, broilers and fattening turkeys). The goal was to provide information on the reservoirs of resistant bacteria that could potentially be transferred between animal populations and from animals to humans [8]. According to the European Union Summary Report on Antimicrobial Resistance in zoonotic bacteria and indicator bacteria from humans, regarding animals and food in 2018/2019 [8], all antimicrobial resistance traits, identified in this study (sulfamethoxazole, tetracycline, chloramphenicol, ampicillin, ciprofloxacin and nalidixic acid), were already highly disseminated among food-producing animals, reinforcing the importance of a One Health approach to better monitor and control public health threats. It is also important to highlight that the three AMR *Escherichia coli* isolates belonged to serotypes already found in confirmed cases of *E. coli* human infections [53,54,55] and that the MDR isolate belonged to serogroup O145, which was in the “top five” most frequent serogroups reported in confirmed cases of human STEC infections in EU/EEA in 2020 [56].

Regarding serotyping and genotypic characterization of the isolated *Salmonella*, and its potential relation with human infection, it is important to notice that *Salmonella* Enteritidis, one of the identified serotypes, is among the most prevalent foodborne pathogen worldwide [57]. ST11, the one found in this study, is the major *Salmonella* Enteritidis ST and has been associated with recent multi-country outbreaks in Europe [58,59]. Although, according to the diversity in Enterobase (as of August 2022), *Salmonella* Schleissheim has been detected in humans, cattle, poultry, wild animals, foodstuffs and environmental samples, the *S*. Schleissheim isolated from wild animals (wild boar and red deer), in the present study, belonged to ST53, which, thus far, has only been reported in foodstuffs (https://enterobase.warwick.ac.uk/species/index/senterica (accessed on 6 October 2022)). *Salmonella* Veneziana serotype, isolated here from a hedgehog, has been considered a non-significant serotype in human disease. However, according to the diversity in Enterobase (as of August 2022), it has already been isolated from humans (https://enterobase.warwick.ac.uk/species/index/senterica (accessed on 6 October 2022)) and has already been potentially associated with a case of acute terminal Ileitis [60].

Concerning the isolated *Campylobacter* species, *C. coli*, identified in one genet sample, is the second most frequently reported *Campylobacter* species in human infections [61]. The identified sequence type, ST1595, is documented (based on the PubMLST collection as of August 2022) to have already been found in isolates from humans with campylobacteriosis, pigs, poultry (broilers and turkeys), waters, and other environmental samples (https://pubmlst.org/organisms/campylobacter-jejunicoli (accessed on 6 October 2022)). *Campylobacter hyointestinalis* serovar, isolated here from 7 wild boars, has already been isolated from several different animal species and is a member of the “emerging *Campylobacter* spp.” group that can also cause disease in humans [62].

With reference to *E. coli* typing results, it is relevant to mention that three of the identified O antigens belong to the top 20 most frequent serogroups reported in confirmed cases of human STEC infections in EU/EEA, 2015–2017 (O145, O146, O121) [63]. Moreover, serotype O26:H11, which has emerged as one of the most common non-O157 STEC strains causing human diseases in many countries [64], was detected in a fallow deer within this study. Contrarily to the isolates found in humans, the isolate collected in this study lacked Stx toxin genes. The absence of Stx-harboring phages in *E. coli* O26:H11 has already been found in isolates from healthy cattle and sheep at slaughter in Switzerland [65]. This genome alteration may promote *E. coli* resilience outside the host and enable adaptation to stress conditions in the gastrointestinal tract [66]. As in this study, serotypes O146:H28 and O27:H30 were already found in *E. coli* isolates in fecal samples from Iberian Peninsula ungulates [11,15]. In the scope of our work, as previously stated by Dias et al. [15], O27:H30 serotype was recovered from fecal samples of both deer and wild boar, confirming that the previously described association of this serotype with deer [11] no longer exists.

Looking at the toxin genes identified on the 52 pathogenic *E. coli* isolates, in agreement with other European studies performed on wild ungulates [15,16,41,67], our results showed that *stx*2 had a higher prevalence (71.2%) than *stx*1 (21.2%) among pathogenic *E. coli* isolates. Only 11 of the 37 STEC isolates contained both *stx*1 and stx2 genes, and none of them contained only *stx*1 gene. Although clinical epidemiological studies suggest that *stx2* is more often associated with severe disease and development of hemolytic–uremic syndrome (HUS) than *stx1*, pointing out for a high pathogenicity of the isolated STECs, the *stx2* subtype found in this study was always *stx2b,* a subtype with a potency similar to that of *stx1* [68].

Analysis of STEC in Europe showed that stx2b, alone, or together with stx1c, is common in STEC from deer dropping and wildlife populations [9] but does not appear to cause severe human illness. Nevertheless, some studies have reported that 10 to 15% of human clinical samples from diarrheal illnesses are positive for stx1c and/or stx2b [69,70].

Heat-stable enterotoxin b gene (*stb*), which is known to be mainly found in association with porcine ETEC [71], was also only detected in the wild boar ETEC isolates (Table 2), one of them also showing the presence of heat-stable enterotoxin a (*sta1*) gene.

Two of the most common *E. coli* STs detected (442 and 738), were previously associated with cases of human disease. ST442 has been significantly associated with hemolytic uremic syndrome [72] and ST738 was one of the STs isolated during 2010–2014 from human cases of infection in Switzerland [70].

Regarding cgMLST analysis of the 52 *E. coli* pathogenic isolates, it is noteworthy that nine genetic clusters were detected from which six enrolled isolates collected from different animal species, evidencing direct or indirect transmission of the *E. coli* isolates between animals cohabiting in the studied natural conservation center. This was expected, since the TNM ungulates live in a geographically limited area with high animal population density. Moreover, there are several drinking fountains shared by these animals, and, in the summer, there is a food supplementation program that brings the animals that inhabit TNM closer together, thus, likely increasing the contact rate.

Another important observation regarded a genetic cluster (cluster_9) that comprised two nearly isogenic *E. coli* isolates with different antibiotic-resistance phenotypes (susceptible vs. MDR) (Table 3), a feature possibly acquired by horizontal gene transfer. One cannot discard the hypothesis that the circulation and transmission of AMR determinants might be linked to the proximity between humans and the studied population during visits and hunting activities. The river that crosses TNM may also be a wildlife–livestock–human interface to take in consideration. In fact, during this study, three samples from TNM river water were also analyzed and it is important to highlight the presence, in one of the samples, of an O157:H7, ST11 *E. coli* strain, which is known to be the most relevant pathogenic *E. coli* in humans. This isolate was susceptible to all tested antibiotics. All the other samples tested negative for pathogenic *E. coli*. 

## 5. Conclusions

In conclusion, we can say that a wide range of factors, related to the transmission and ecology of diseases (namely increasing pressure of humans on natural ecosystems and rising interactions between the different species), has reached a plateau without precedent and this is a major concern for the control of wildlife diseases. Animal health surveillance is recognized as a key element in preventing public health risks related to emerging zoonotic diseases. From a public health perspective, the present research highlights and confirms that wild animals constitute important reservoirs of zoonotic pathogens like *Escherichia coli*, *Salmonella* spp. and *Campylobacter* spp., including resistant and MDR strains. These results reinforce the importance of a One Health approach, showing that a better understanding of community ecology is essential for a better understanding of the epidemiological links between all actors in the wildlife–livestock–human continuum. To our best knowledge, this is the first study in Portugal, and one of the few in Europe, that simultaneously performs the phenotypic and genotypic characterizations of three of the most common foodborne bacteria (*Campylobacter* spp., *Salmonella* spp., and pathogenic *E. coli*) isolated in fecal samples from wild animals.

## Figures and Tables

**Table 1:** Isolation and characterization of STEC and non-STEC *E. coli*, *Salmonella* spp. and *Campylobacter* spp. in 132 tested samples.

		TNM			CRFPBG		Total
		Wild Boar	Fallow Deer	Red Deer	Hedgehog	Genet	
**Samples tested for *E. coli***		51	50	12	18	1	132
*E. coli* isolates	STEC No. (% +ve samples)	3 (5.9)	29 (56.0 ^a^)	5 (41.7)	0	0	37 (27.3 ^a^)
	EPEC No. (% +ve samples)	3 (5.9)	7 (14.0)	1 (8.3)	1 (5.6)	0	12 (9.1)
	ETEC No. (% +ve samples)	3 (5.9)	0	0	0	0	3 (2.3)
	Total No. (% +ve samples)	9 (17.6)	36 (68.0 ^a^)	6 (50.0)	1 (5.5)	0	52 (37.9 ^a^)
**Samples tested for *Salmonella* spp.**		51	50	12	18	1	132
*Salmonella* isolates	*S.* Enteritidis No. (% + ve samples)	1 (2.0)	0	0	0	0	1 (0.8)
	*S.* Schleissheim No. (% +ve samples)	1 (2.0)	0	1 (8.3)	0	0	2 (1.5)
	*S*. Veneziana No. (% +ve samples)	0	0	0	1 (5.6)	0	1 (0.8)
	Total No. (% +ve samples)	2 (3.9)	0	1 (8.3)	1 (5.6)	0	4 (3.0)
**Samples tested for *Campylobacter* spp.**		39	50	12	16	1	118 ^b^
*Campylobacter* isolates	*C. hyointestinalis* No. (% +ve samples)	7 (17.9)	0	0	0	0	7 (5.9)
	*C. coli* No. (% +ve samples)	0	0	0	0	1 (100)	1 (0.8)
	Total No. (% +ve samples)	7 (17.9)	0	0	0	1 (100)	8 (6.8)

TNM, Tapada Nacional de Mafra; CRFPBG, Centro de Recuperação de Fauna do Parque Biológico de Gaia; STEC, Shiga toxin-producing *E. coli*; EPEC, Enteropathogenic *E. coli*; ETEC, Enterotoxigenic *E. coli*; No., Number; +ve, Positive. ^a^ Different pathogenic *E. coli* were detected in the same sample from one fallow deer, one isolate identified as EPEC and the other as STEC, and two different STEC isolates were detected in other fallow deer; ^b^ 14 samples did not have enough material for *Campylobacter* detection.

**Table 2:** Distribution of serotypes, sequence types, pathotypes and virulence determinants among the 52 pathogenic *E. coli* isolates, determined by PCR and WGS.

**O Antigen**		O146		O75	O27	OND	O110	OND	OND	O121	O156	O70	O108	O145	O26	O35	O167	OND	O28ac/O42	O98	O182	**Total (%)**
**H Antigen**		H21	H28	H8	H30	H8	H31	H21	H28	HND	H25	H11	H9	HND	H11	H31	H9	HND	H8	H5	H19	
**ST**		442	738	13	753	13	812	11692-like	738	655	300	29	302	137	29	123	2538	137	109	2951	10	
**Pathotype**		**STEC**								**EPEC**									**ETEC**			
No. isolates		10	8	10	5	1	1	1	1	3	2	1	1 *	1 **	1	1	1	1	1 *	1	1	52
Animals	Wild boar	2	0	0	1	0	0	0	0	0	0	1	0	1	0	0	1	0	1	1	1	9 (17.3)
	Fallow deer	8	7	10	2	1	0	1	0	2	2	0	1	0	1	0	0	1	0	0	0	36 (69.2)
	Red deer	0	1	0	2	0	1	0	1	1	0	0	0	0	0	0	0	0	0	0	0	6 (11.5)
	Hedgehog	0	0	0	0	0	0	0	0	0	0	0	0	0	0	1	0	0	0	0	0	1 (1.9)
Toxin	*astA*	0	8	0	5	0	0	0	1	3	2	1	0	1	1	1	1	1	0	1	0	26 (50.0)
	*ehxa*	9	8	10	0	1	1	1	1	3	2	1	0	1	1	0	0	1	0	0	0	40 (76.9)
	*mchF*	10	8	0	5	0	1	1	1	0	0	0	1	0	0	0	0	0	0	0	0	27 (51.9)
	*sta1*	0	0	0	0	0	0	0	0	0	0	0	0	0	0	0	0	0	0	0	1	1 (1.9)
	*sTb*	0	0	0	0	0	0	0	0	0	0	0	0	0	0	0	0	0	1	1	1	3 (5.8)
	*stx1*	0	0	10	0	1	0	0	0	0	0	0	0	0	0	0	0	0	0	0	0	11 (21.2)
	*stx2*	10	8	10	5	1	1	1	1	0	0	0	0	0	0	0	0	0	0	0	0	37 (71.2)
	*subA*	8	7	8	4	1	1	1	1	0	0	0	0	0	0	0	0	0	0	0	0	31 (59.6)
Adhe	*eae*	0	0	0	0	0	0	0	0	3	2	1	1	1	1	1	1	1	0	0	0	12 (23.07)
	*iha*	10	6	10	5	1	1	1	1	2	0	1	0	1	1	0	0	1	0	0	1	42 (80.8)
	*lpfA*	10	7	10	0	1	0	1	1	3	2	1	0	0	1	0	1	0	1	0	0	39 (75.0)
Other	*elt*	0	0	0	0	0	0	0	0	0	0	0	0	0	0	0	0	0	1	1	1	3 (5.8)
	*espl*	10	0	10	2	1	0	1	0	0	0	1	0	0	0	0	0	0	0	0	0	25 (48.1)
	*gad*	10	8	10	1	1	1	1	1	3	2	1	1	1	1	1	1	1	1	0	1	47 (90.4)
	*ireA*	10	8	10	5	1	1	1	1	0	0	0	0	0	0	0	0	0	0	0	0	37 (71.2)
	*iss*	10	7	8	5	1	0	1	1	0	0	1	1	1	1	0	1	1	0	1	0	40 (76.9)
	*ompT*	10	8	8	5	1	1	1	1	3	2	1	1	1	1	1	1	1	0	0	0	47 (90.4)

* Antimicrobial resistant (AMR) isolate; ** Multidrug resistant (MDR) isolate; STEC, Shiga toxin-producing *E. coli*; EPEC, Enteropathogenic *E. coli*; ETEC, Enterotoxigenic *E. coli;* ST, Sequence type; HND, H antigen not determined; OND, O antigen not determined.

**Table 3:** Genetic clusters identified among pathogenic *E. coli* through cgMLST analyses.

Global cgMLST Analysis (*n* = 2567 loci) ^a^									ST-Specific cgMLST Analysis ^b^	
Cluster	Cluster Length	Isolates	Animal Species	Timespan (Days)	Pathotype	MLST_ST	Serotype	Antibiotic Resistance Phenotype	Extended Schema Size	Cluster Confirmation
cluster_5	7	Ec-TM1,Ec-TM28,Ec-TM29-4,Ec-TM31,Ec-TM56,Ec-TM87,Ec-TM92	fallow deer (100.0%)	120	STEC	13	O75:H8 (71.4%), OND:H8 (28.6%)	Susceptible (100.0%)	3321	Yes. Cluster isolates connected by ≤ 9 ADs (0.27%)
cluster_7	2	Ec-TM15,Ec-TM74	fallow deer (100.0%)	112	STEC	13	O75:H8 (100.0%)	Susceptible (100.0%)	3321	Yes. Cluster isolates differ by 11 ADs (0.33%)
cluster_9	2	Ec-TM26,Ec-TM69	fallow deer (50.0%), wild boar (50.0%)	112	EPEC	137	O145:HND (50.0%), OND:HND (50.0%)	AMP, CHL, SMX (50.0%), Susceptible (50.0%)	3333	Yes. Cluster isolates differ by 5 ADs (0.15%)
cluster_3	2	Ec-TM30-2,Ec-TM101	fallow deer (100.0%)	106	EPEC	300	O156:H25 (100.0%)	Susceptible (100.0%)	3387	Yes. Cluster isolates differ by 5 ADs (0.15%)
cluster_6	6	Ec-TM2,Ec-TM3,Ec-TM23,Ec-TM73,Ec-TM82,Ec-TM95	fallow deer (83.3%), wild boar (16.7%)	120	STEC	442 ^c^	O146:H21 (100.0%)	Susceptible (100.0%)	3248	Yes, consolidating the potential link with cluster_4 (12 ADs/0.37%). Ec-TM95 slightly split apart (15 ADs/0.46%)
cluster_4	4	Ec-TM27,Ec-TM84,Ec-TM97,Ec-TM99	fallow deer (75.0%), wild boar (25.0%)	106	STEC	442 ^c^	O146:H21 (100.0%)	Susceptible (100.0%)	3248	Yes, consolidating the potential link with cluster_6 (12 ADs/0.37%). Ec-TM99 slightly split apart (12 ADs/0.37%)
cluster_1	3	Ec-TM14,Ec-TM55,Ec-TM64	fallow deer (66.7%), red deer (33.3%)	105	EPEC	655	O121:HND (100.0%)	Susceptible (100.0%)	3349	Yes. Cluster isolates connected by 5 ADs (0.15%)
cluster_2	2	Ec-TM25,Ec-TM30-1	red deer (50.0%), fallow deer (50.0%)	7	STEC	738	O146:H28 (50.0%), OND:H28 (50.0%)	Susceptible (100.0%)	3334	Yes. Cluster isolates differ by 7 ADs (0.21%)
cluster_8	2	Ec-TM81,Ec-TM96	fallow deer (50.0%), wild boar (50.0%)	1	STEC	753	O27:H30 (100.0%)	Susceptible (100.0%)	3208	Yes. Cluster isolates differ by 8 ADs (0.25%).

^a^ cgMLST analysis based on the core-genome of the 52 isolates (i.e., a sub-schema of the original 7601-loci wgMLST schema comprising loci called in all samples); ^b^ Dynamic cgMLST analysis based on ST-specific core-genome schema (i.e., a sub-schema comprising the loci called in all the isolates of the same ST); ^c^ Given the genetic proximity of the isolates previously identified as ST442-like and ST11692-like (Figure 1), the ST11692-like isolate was included in the analysis of the ST442.

## Data Availability

All supporting data and protocols have been provided within the article or through supplementary data files. Supplementary Material is available with the online version of this article.

## Supplementary Materials

The following supporting information can be downloaded at: https://www.mdpi.com/article/10.3390/microorganisms10112132/s1, Table S1: Primers and PCR profile for detection of *E. coli* virulence genes and Shiga toxins; Table S2: Whole-genome sequencing data of STEC and non-STEC *E. coli*, *Salmonella* spp. and *Campylobacter* spp. isolates.
