# Supplementary material for: Pathogenic Escherichia coli, Salmonella spp. and Campylobacter spp. in Two Natural Conservation Centers of Wildlife in Portugal: Genotypic and Phenotypic Characterization
Source: Microorganisms. 2022 Oct 27;10(11):2132. doi: 10.3390/microorganisms10112132 (PMC9694878; doi:10.3390/microorganisms10112132)
Supplement: Supplementary file 1 [file microorganisms-10-02132-s001.zip › microorganisms-1986376-supplementary/microorganisms-1986376-suppl-proofback/Table S1.pdf]

Target gene	Primer sequence (5'-3')	Thermal profile	Amplicon size (pb)	Reference
eae	GGYCAGCGTTTTTTCCTTCCTG<br>TCGTCACCARAGGAATCGGAG	95ºC 15 min<br>35x: 94ºC 50 sec, 55.5ºC 40 sec, 72ºC 1 min<br>72ºC 3 min<br>4ºC ∞	377	Persson 2007
aggR	CATTCTTGATTGCATAAGGATCTGG<br>GCAATCAGATTAARCAGCGATACA		426	Nadia 2012
eltI	AACGTTCCGGAGGTCTTATG<br>CAACCTTGTGGTGCATGATG		511	Fujioka 2013
estp	ACTGAATCACTTGACTCTTCA<br>TCACAGCAGTAAAATGTGTTGT		120	
esth	TTCACCTTTCCCTCAGGATG<br>ATAGCACCCGGTACAAGCAG		172	
ipaH	TTGACCGCCTTTCCGATACC<br>ATCCGCATCACCGCTCAGAC		647	Persson 2007
stx1	GTACGGGGATGCAGATAAATCGC<br>AGCAGTCATTACATAAGAACGYCCACT	95ºC 15 min<br>35x: 94ºC 50 sec, 60ºC 40 sec, 72ºC 1 min<br>72ºC 3 min<br>4ºC ∞	206	Scheutz 2012
stx2	GGCACTGTCTGAAACTGCTCCTGT<br>ATTAAACTGCACTTCAGCAAATCC<br>CGCTGTCTGAGGCATCTCCGCT<br>TAAACTTCACCTGGGCAAAGCC		627	

eae , intimin-coding gene*; aggR* , gene encoding a transcriptional regulator of enteroaggregative *E. coli; eltI* , eat labile toxin coding gene; *estp* , gene encoding the heat stable enterotoxin A (porcine variant); *esth* , gene encoding the heat stable enterotoxin A (human variant)*; ipaH* , invasion plasmid antigen H gene; *stx,* Stx-coding gene. Primers: R = A or G; Y = C or T.
