# Supplementary material for: Pathogenic Escherichia coli, Salmonella spp. and Campylobacter spp. in Two Natural Conservation Centers of Wildlife in Portugal: Genotypic and Phenotypic Characterization
Source: Microorganisms. 2022 Oct 27;10(11):2132. doi: 10.3390/microorganisms10112132 (PMC9694878; doi:10.3390/microorganisms10112132)
Supplement: Supplementary file 1 [file microorganisms-10-02132-s001.zip › microorganisms-1986376-supplementary/microorganisms-1986376-suppl-proofback/Table S2.pdf]

Isolate ID	Year/Month	Animal	Pathotype	AMR phenotype	Serotype	Virulence genes	Resistance genes	Plasmids	MLST	Genetic cluster	Sample ID	Ena Acession #
E. coli												
Ec-TM1	2020/07	Fallow deer	STEC	Susceptible	O75:H8	*cba, cma, ehxA, espI, gad, iha, ireA, iss, IpfA, ompT, senB, stx1c, stx2b, subA, terC, traT*	No	-	13	5	PT_EC0001	ERS12460637
Ec-TM2	2020/07	Fallow deer	STEC	Susceptible	O146:H21	*cba, cma, ehxA, espI, gad, iha, ireA, iss, iucC, iutA, IpfA, mchB, mchC, mchF, ompT, senB, stx2b, subA, terC, traT*	No	-	442	6	PT_EC0002	ERS12460638
Ec-TM3	2020/07	Fallow deer	STEC	Susceptible	O146:H21	*cba, cma, ehxA, espI, gad, iha, ireA, iss, iucC, iutA, IpfA, mchB, mchC, mchF, ompT, senB, stx2b, subA, terC, traT*	No	-	442	6	PT_EC0003	ERS12460639
EC-TM14	2020/07	Red deer	EPEC	Susceptible	O121:HND	*eae, astA, celb, ehxA, espA, espB, espF, espJ, etpD, gad, iha, IpfA, nleA, nleB, nleC, ompT, tccP, terC, tir, toxB, traT*	No	-	655	1	PT_EC0004	ERS12460640
EC-TM15	2020/07	Fallow deer	STEC	Susceptible	O75:H8	*cba, cma, ehxA, espI, gad, iha, ireA, iucC, iutA, IpfA, senB, stx1c, stx2b, subA, terC, traT*	No	-	13	7	PT_EC0005	ERS12460641
EC-TM17	2020/07	Wild boars	ETEC	TET, SMX	O28ac/O42:H8	*f17A, f17G, gad, hra, IpfA, stb, trC*	*tet(B) , sul2*	Not found	109	-	PT_EC0006	ERS12460642
Ec-TM22	2020/07	Fallow deer	STEC	Susceptible	O146:H28	*astA, ehxA, gad, hra, iha, ireA, iss, IpfA, ompT, subA, mchB, mchC, mchF, stx2b, terC, traT, usp*	No	-	738	-	PT_EC0007	ERS12460643
Ec-TM23	2020/07	Fallow deer	STEC	Susceptible	O146:H21	*cba, cma, ehxA, espI, gad, iha, ireA, iss, iucC, iutA, IpfA, mchB, mchC, mchF, ompT, senB, stx2b, subA, terC, traT*	No	-	442	6	PT_EC0008	ERS12460644
Ec-TM24	2020/07	Red deer	STEC	Susceptible	O146:H28	*astA, ehxA, gad, hra, iha, ireA, iss, IpfA, mchB, mchC, mchF, ompT, stx2b, subA, terC, traT, usp*	No	-	738	-	PT_EC0009	ERS12460645
Ec-TM25	2020/07	Red deer	STEC	Susceptible	OND:H28	*astA, ehxA, gad, hra, iha, ireA, iss, IpfA, mchB, mchC, mchF, ompT, stx2b, subA, terC, traT, usp*	No	-	738	7	PT_EC0010	ERS12460646
Ec-TM26	2020/07	Wild boars	EPEC	AMP, CHL, SMX	O145:HND	*astA, cea, chA, cif, eae, ehxA, espA, espB, espF, espJ, etpD, gad, iha, iss, iucC, iutA, katP, neuC, nleA, nleB, nleC, ompT, tccP, terC, tir, toxB, traT*	*blaTEM-1B, floR, sul2*	IncFII (100%), IncFIB (96.92%)	137	9	PT_EC0011	ERS12460647
Ec-TM27	2020/07	Fallow deer	STEC	Susceptible	O146:H21	*cba, cma, ehxA, espI, gad, iha, ireA, iss, IpfA, mchB, mchC, mchF, ompT, stx2b, sub2, terC, traT*	No	-	442	4	PT_EC0012	ERS12460648
Ec-TM28	2020/07	Fallow deer	STEC	Susceptible	O75:H8	*cba, cma, ehxA, espI, gad, iha, ireA, iss, IpfA, ompT, senB, stx1c, stx2b, subA, terC, traT*	No	-	13	5	PT_EC0013	ERS12460649
Ec-TM29-2	2020/07	Fallow deer	STEC	Susceptible	O146:H28	*astA, ehxA, gad, hra, iha, ireA, iss, mchB, mchC, mchF, ompT, stx2b, subB, terC, traT, usp*	No	-	738	-	PT_EC0014	ERS12460650
Ec-TM29-4	2020/07	Fallow deer	STEC	Susceptible	OND:H8	*cba, cma, ehxA, espI, gad, iha, ireA, iss, IpfA, ompT, senB, stx1c, stx2b, subA, terC, traT*	No	-	13	5	PT_EC0015	ERS12460651
Ec-TM30-1	2020/07	Fallow deer	STEC	Susceptible	O146:H28	*astA, ehxA, gad, hra, iha, ireA, iss, IpfA, mchB, mchC, mchF, ompT, stx2b, subA, terC, traT, usp*	No	-	738	7	PT_EC0017	ERS12460653
Ec-TM30-2	2020/07	Fallow deer	EPEC	Susceptible	O156:H25	*astA, eae, ehxA, espA, espF, espJ, espP, gad, IpfA, nleA, nleB, ompT, terC, tir, traT*	No	-	300	3	PT_EC0016	ERS12460652
Ec-TM31	2020/07	Fallow deer	STEC	Susceptible	O75:H8	*cba, cma, ehxA, espI, gad, iha, ireA, iss, iIpfA, ompT, semB, stx1c, stx2b, subA, terC, traT*	No	-	13	5	PT_EC0018	ERS12460654
Ec-TM36	2020/07	Wild boars	ETEC	Susceptible	O98:H5	*astA, fyuA, irp2, iss, stb, terC, traT*	No	-	2951	-	PT_EC0019	ERS12460655
Ec-TM38	2020/07	Red deer	STEC	Susceptible	O110:H31	*chuA, exhA, fyuA, gad, iha, ireA, irp2, kpsE, mchF, ompT, stx2, subA, terC, traT, vat, yfcV*	No	-	812	-	PT_EC0020	ERS12460656
Ec-TM42	2020/10	Fallow deer	STEC	Susceptible	O146:H28	*astA, ehxA, gad, hra, iha, ireA, iss, IpfA, mchB, mchC, mchF, ompT, stx2b, subA, terC, traT, usp*	No	-	738	-	PT_EC0021	ERS12460657
Ec-TM47	2020/10	Wild boars	ETEC	Susceptible	O182:H19	*capU, fyupA, gad, iha, irp2, sitA, sta1, stb, terC, traT*	No	-	10	-	PT_EC0022	ERS12460658
Ec-TM54	2020/10	Fallow deer	STEC	Susceptible	OND:H21	*cba, cma, ehxA, espI, gad, iha, ireA, iss, iucC, iutA, IpfA, mchB, mchC, mchF, ompT, stx2b, subA, terC, traT*	No	-	11692-like	-	PT_EC0023	ERS12460659
Ec-TM55	2020/10	Fallow deer	EPEC	Susceptible	O121:HND	*astA, celb, eae, ehxA, espA, espB, espF, espJ, etpD, gad, IpfA, nleA, nleB, nleC, ompT, tccP, terC, tir, toxB, traT*	No	-	655	1	PT_EC0024	ERS12460660
Ec-TM56	2020/10	Fallow deer	STEC	Susceptible	O75:H8	*cba, cma, ehxA, espI, gad, iha, ireA, iss, IpfA, ompT, senB, stx1c, stx2b, subA, terC, traT*	No	-	13	5	PT_EC0025	ERS12460661
Ec-TM57	2020/10	Wild boars	EPEC	Susceptible	O70:H11	*astA, cba, cif, cma, eae, ehxA, espA, espB, espF, espI, espJ, etpD, fyuA, gad, iha, irp2, iss, iucC, iutA, IpfA, nleA, nleB, ompT, tccP, terC, tir, traT*	No	-	29	-	PT_EC0026	ERS12460662
Ec-TM61	2020/10	Red deer	STEC	Susceptible	O27:H30	*astA, celb, chuA, eilA, iha, ireA, iss, mchB, mchC, mchF, ompT, stx2b, subA, terC, traT*	No	-	753	-	PT_EC0027	ERS12460663
Ec-TM64	2020/10	Fallow deer	EPEC	Susceptible	O121:HND	*astA, celb, eae, ehxA, espA, espB, espF, espJ, etpD, gad, iha, IpfA, nlea, nleB, nleC, ompT, tccP, terC, tir, toxB, traT*	No	-	655	1	PT_EC0028	ERS12460664
Ec-TM65	2020/10	Fallow deer	EPEC	Susceptible	O26:H11	*astA, cba, cif, cma, eae, efa1, ehxA, espA, espB, espF, espJ, etpD, fyuA, gad, iha, irp2, iss, iucC, iutA, IpfA, nleA, nleB, ompT, tccP, terC, tir, traT*	No	-	29	-	PT_EC0029	ERS12460665
Ec-TM66	2020/10	Red deer	STEC	Susceptible	O27:H30	*astA, celb, chuA, eilA, espI, iha, ireA, iss, mchB, mchC, mchF, ompT, stx2b, subA, terC, traT*	No	-	753	-	PT_EC0030	ERS12460666
Ec-TM67	2020/11	Wild boars	EPEC	Susceptible	O167:H9	*astA, cif, eae, eilA, espA, espB, espJ, gad, iss, IpfA, nleA, ompT, terC, tir*	No	-	2538	-	PT_EC0031	ERS12460667
Ec-TM69	2020/11	Fallow deer	EPEC	Susceptible	OND:HND	*astA, cea, chuA, cif, eae, ehxA, espA, espB, espF, espJ, etpD, gad, iha, iss, iucC, iutA, katP, neuC, nleA, nleB, nleC, ompT, tccP, terC, tir, traT*	No	-	137	9	PT_EC0032	ERS12460668
Ec-TM71	2020/11	Fallow deer	STEC	Susceptible	O75:H8	*cba, cma, ehxA, espI, gad, iha, ireA, iss, IpfA, ompT, senB, stx1c, stx2b, terC, traT*	No	-	13	-	PT_EC0033	ERS12460669
Ec-TM72	2020/11	Fallow deer	STEC	Susceptible	O146:H28	*astA, ehxA, gad, hra, iha, ireA, iss, IpfA, mchB, mchC, mchF, ompT, stx2b, subA, terC traT, usp*	No	-	738	-	PT_EC0034	ERS12460670
Ec-TM73	2020/11	Fallow deer	STEC	Susceptible	O146:H21	*cba, cma, ehxA, espI, gad, iha, ireA, iss, iucC, iutA, IpfA, mchB, mchC, mchF, ompT, senB, stx2b, subA, terC, traT*	No	-	442	6	PT_EC0035	ERS12460671
Ec-TM74	2020/11	Fallow deer	STEC	Susceptible	O75:H8	*cba, cma, ehxA, espI, gad, iha, ireA, iucC, iutA, IpfA, senB, stx1c, stx2b, subA, terC, traT*	No	-	13	7	PT_EC0036	ERS12460672
Ec-TM81	2020/11	Wild boars	STEC	Susceptible	O27:H30	*astA, chuA, ellA, gad, iha, ireA, iss, mchB, ,mchC, mchF, ompT, stx2b, terC, traT*	No	-	753	8	PT_EC0037	ERS12460673
Ec-TM82	2020/11	Wild boars	STEC	Susceptible	O146:H21	*cba, cma, ehxA, espI, gad, iha, ireA, iss, iucC, iutA, IpfA, mchB, mchC, mchF, ompT, senB, stx2b, terC, traT*	No	-	442	6	PT_EC0038	ERS12460674
Ec-TM84	2020/11	Wild boars	STEC	Susceptible	O146:H21	*cba, cma, espI, gad, iha, ireA, iss, iucC, iutA, IpfA, mchB, mchC, mchF, ompT, stx2b, subA, terC, traT*	No	-	442	4	PT_EC0039	ERS12460675
Ec-TM86	2020/11	Fallow deer	STEC	Susceptible	O27:H30	*astA, chuA, eilA, espI, fyuA, iha, ireA, iss, mchB mchC, mchF, ompT, stx2b, subA, terC, traT*	No	-	753	-	PT_EC0040	ERS12460676
Ec-TM87	2020/11	Fallow deer	STEC	Susceptible	O75:H8	*cba, cma, ehxA, espI, gad, iha, ireA, iss, IpfA, ompT, senB, stx1c, stx2b, subA, terC, traT*	No	-	13	5	PT_EC0041	ERS12460677
Ec-TM89	2020/11	Fallow deer	STEC	Susceptible	O146:H28	*astA, ehxA, gad, hra, iha, ireA, iss, IpfA, mchB, mchC, mchF, ompT, stx2b, subA, terC, traT, usp*	No	-	738	-	PT_EC0042	ERS12460678
Ec-TM91	2020/11	Fallow deer	STEC	Susceptible	O146:H28	*astA, ehxA, gad, hra, iha, ireA, IpfA, mchB, mchC, mchF, ompT, stx2b, subA, terC, traT, usp*	No	-	738	-	PT_EC0043	ERS12460679
Ec-TM92	2020/11	Fallow deer	STEC	Susceptible	O75:H8	*cba, cma, ehxA, espI, gad, iha, ireA, iss, IpfA, ompT, senB, stx1c, stx2b, subA, terC, traT*	No	-	13	5	PT_EC0044	ERS12460680
Ec-TM95	2020/11	Fallow deer	STEC	Susceptible	O146:H21	*cba, cma, ehxA, espI, gad, iha, ireA, iss, iucC, iutA, IpfA, mchB, mchC, mchF, ompT, senB, stx2b, subA, terC, traT*	No	-	442	6	PT_EC0045	ERS12460681
Ec-TM96	2020/11	Fallow deer	STEC	Susceptible	O27:H30	*astA, celb, chuA, eilA, iha, ireA, iss, mchB, mchC, mchF, ompT, stx2b, subA, terC, traT*	No	-	753	8	PT_EC0046	ERS12460682
Ec-TM97	2020/11	Fallow deer	STEC	Susceptible	O146:H21	*cba, cma, ehxA, espI, gad, iha, ireA, iss, iucC, iutA, IpfA, mchB, mchC, mchF, ompT, stx2b, subA, terC, traT*	No	-	442	4	PT_EC0047	ERS12460683
Ec-TM98	2020/11	Fallow deer	STEC	Susceptible	O75:H8	*cba, cma, ehxA, espI, gad, iha, ireA, iss, IpfA, ompT, senB, stx1c, stx2b, subA, terC, traT*	No	-	13	-	PT_EC0048	ERS12460684
Ec-TM99	2020/11	Fallow deer	STEC	Susceptible	O146:H21	*cba, cma, ehxA, espI, gad, iha, ireA, iss, iucC, iutA, lpfA, mchB, mchC, mchF, ompT, stx2b, subA, terC, traT*	No	-	442	4	PT_EC0049	ERS12460685
Ec-TM100	2020/11	Fallow deer	EPEC	AMP, NAL, CIP	O108:H9	*cea, chuA, cia, eae, espA, espB, espJ, gad, iss, mchF, neuC, nleC, ompT, terC, tir, traT*	*blaTEM-1D, qnrB36, qnrB19, qnrB82, gyrA*	Col (100%), IncFII (100%), IncX1 (98.94%), p0111 (98.98%)	302	-	PT_EC0050	ERS12460686
Ec-TM101	2020/11	Fallow deer	EPEC	Susceptible	O156:H25	*astA, eae, ehxA, espA, espF, espJ, espP, etpD, gad, IpfA, nleA, nleB, ompT, terC, tir, traT*	No	-	300	3	PT_EC0051	ERS12460687
Ec-PBG1	2020/11	Hedgehog	EPEC	Susceptible	O35:H31	*astA, chuA, cif, eae, efa1, espA, fyuA, gad, ibeA, irp2, nleB, nleC, ompT, sitA, terC, tir, traT, usp, yfcV*	No	-	123	-	PT_EC0068	ERS12460704

Isolate ID	Year/Month	Animal	Serotype	AMR phenotype	MLST	Resistance genes	Plasmids	Sample ID	Ena Acession #
Salmonella enterica									
Se-TM77	2020/11	Red deer	Schleissheim	Susceptible	53	*aac(6')-Iaa*	Not found	PT_SE0144	ERS12843885
Se-TM79	2020/11	Wild boars	Enteritidis	Susceptible	11	*aac(6')-Iaa*	IncFII (100%), IncFIB (98.91%)	PT_SE0145	ERS12843886
Se-TM83	2020/11	Wild boars	Schleissheim	Susceptible	53	*aac(6')-Iaa*	Not found	PT_SE0146	ERS12843887

Isolate ID	Year/Month	Animal	Species	AMR phenotype	MLST	Resistance genes	Sample ID	Ena Acession #
Campylobacter								
Cc_PBG26	2021/01	Geneta	*C. coli*	AMP, CIP, TET	1595	*blaOXA-61, gyrA, tetO*	PT_CC_PBG26	ERS13408374
